# Up-Regulation of Immune Checkpoints in the Thymus of PRRSV-1-Infected Piglets in a Virulence-Dependent Fashion

**DOI:** 10.3389/fimmu.2021.671743

**Published:** 2021-05-11

**Authors:** Inés Ruedas-Torres, Irene M. Rodríguez-Gómez, José María Sánchez-Carvajal, Silvia Guil-Luna, Fernanda Larenas-Muñoz, Francisco J. Pallarés, Librado Carrasco, Jaime Gómez-Laguna

**Affiliations:** ^1^ Department of Anatomy and Comparative Pathology and Toxicology, International Agrifood Campus of Excellence (ceiA3), Faculty of Veterinary Medicine, University of Córdoba, Córdoba, Spain; ^2^ Maimónides Institute for Biomedical Research of Córdoba, IMIBIC, Córdoba, Spain

**Keywords:** immune checkpoints, thymus, immunosuppression, T-cell exhaustion, PRRSV

## Abstract

Virulent porcine reproductive and respiratory syndrome virus (PRRSV) strains, such as the Lena strain, have demonstrated a higher thymus tropism than low virulent strains. Virulent PRRSV strains lead to severe thymus atrophy, which could be related to marked immune dysregulation. Impairment of T-cell functions through immune checkpoints has been postulated as a strategy executed by PRRSV to subvert the immune response, however, its role in the thymus, a primary lymphoid organ, has not been studied yet. Therefore, the goal of this study was to evaluate the expression of selected immune checkpoints (*PD1/PDL1, CTLA4, TIM3, LAG3, CD200R1* and *IDO1*) in the thymus of piglets infected with two different PRRSV-1 strains. Thymus samples from piglets infected with the low virulent 3249 strain, the virulent Lena strain and mock-infected were collected at 1, 3, 6, 8 and 13 days post-infection (dpi) to analyze PRRSV viral load, relative quantification and immunohistochemical staining of immune checkpoints. *PD1/PDL1*, *CTLA4*, *TIM3*, *LAG3* and *IDO1* immune checkpoints were significantly up-regulated in the thymus of PRRSV infected piglets, especially in those infected with the virulent Lena strain from 6 dpi onwards. This up-regulation was associated with disease progression, high viral load and cell death. Co-expression of these molecules can affect T-cell development, maturation and selection, negatively regulating the host immune response against PRRSV.

## Introduction

Porcine reproductive and respiratory syndrome (PRRS) is caused by PRRS virus (PRRSV), an RNA virus which presents a huge genetic and antigenic variability, being classified into two different viral species, *Betaarterivirus suid-1* (former PRRSV-1) and *Betaarterivirus suid-2* (former PRRSV-2) within the order *Nidovirales* and family *Arteriviridae* (1). PRRSV preferentially infects cells of the monocyte/macrophage lineage, with the porcine alveolar macrophage serving as primary cell target (2). Dendritic cells (DCs) have been also reported to support PRRSV replication *in vitro* (3), which along with alveolar macrophages would act in the first line of the host defense against pathogen invasion. It is known that PRRSV suppresses the innate immunity that leads to inefficient adaptive immune response in infected pigs (4).

The thymus, as primary lymphoid organ, gains special interest in the study of the immunopathogenesis of PRRS, because of its role in T-cell development and maturation (5), but also, for being more affected by virulent PRRSV-1 strains, such as Lena or SU1-bel strains (6, 7). Such strains are capable of inducing high apoptosis rates in the thymic cortex, leading to a severe atrophy of the organ in comparison to low virulent strains (6–8). These findings support the interest of studying the immunopathogenesis of PRRS in the thymus of pigs infected with strains of different virulence.

Immune checkpoint molecules are regulatory receptors expressed on immune cells, which are able to maintain self-tolerance and modulate the breadth of the effector immune responses in peripheral tissues (9, 10). However, during a high number of infectious diseases, up-regulation of these molecules can also limit the severe inflammatory response elicited by the infection (9, 10). Sustained signaling through immune checkpoints causes T-cell dysfunction, driving effector immune cells, especially T-cells, into a state of ‘exhaustion’ (10–12). ‘T-cell exhaustion’ is defined by poor effector functions, persistent expression of inhibitory receptors, such as immune checkpoints, poor recall responses and a transcriptional state distinct from that of functional effector or memory T-cells (10, 12, 13). However, the role of immune checkpoints in viral infectious diseases of pigs has been scarcely studied.

Programmed cell death 1 (*PD1*) and its ligand, programmed cell death ligand (*PDL1*) is the most studied immune checkpoints axis in pigs. An up-regulation of both molecules has been associated *in vivo* with a decrease in T-cell proliferation and the impairment of the immune response during acute classical swine fever virus (CSFV) infection (14). An increased expression of PD-L1, PD-L2 and phosphatase and tensin homologue deleted on chromosome ten (PTEN) has been also reported in pigs suffering from postweaning multisystemic wasting syndrome (PMWS) (15). In a porcine circovirus type 2 (PCV-2) single infection or PCV-2/PRRSV-2 co-infection model, Richmond and co-authors (2015) demonstrated low levels of lymphocyte apoptosis in PD-1 deficient lymphocytes in co-culture with infected monocyte-derived DCs. The same authors reported higher lymphocyte apoptosis rates in lymphocytes expressing normal PD-1 levels, suggesting a potential mechanism involved in lymphocyte depletion (16). Transcriptional analysis of inguinal lymph node from PRRSV-infected pigs also showed an up-regulation of the axis PD-1/PD-L1 and hepatitis A virus cellular receptor (also *TIM3*) (17). Other immune checkpoints, such as cytotoxic T-lymphocyte associated protein 4 (*CTLA4*), lymphocyte activating 3 (*LAG3*) and indoleamine 2,3-dioxygenase 1 (*IDO1*) have been also studied in immunosuppressive viral diseases such as PMWS and CSF (15, 18). However, the role of these and other immune checkpoints described in pigs, such as CD200 receptor 1 (*CD200R1*), remains unclear (17–22).

Although a significant number of investigations into PRRSV immunopathogenesis have been conducted, the role that immune checkpoints may play in the context of this disease still remains unexplored. Therefore, the goal of this study was to evaluate the expression of selected immune checkpoints (*PD1/PDL1, CTLA4, TIM3, LAG3, CD200R1* and *IDO1*) in the thymus of piglets infected with two PRRSV-1 strains (3249 and Lena) of different virulence and to determine their correlation with the disease course during the early stage of infection.

## Material and Methods

### Animals and Experimental Design

This study formed part of a large project performed to investigate the pathogenesis of PRRSV-1 strains of different virulence. Animals and samples were collected from experiments published elsewhere (7, 23). In brief, sixty-five 4-week-old Landrace x Large White piglets were randomly separated in three different barns at the Centre de Recerca en Sanitat Animal (IRTA-CReSA, Cerdanyola del Vallès, Barcelona, Spain). All pigs were negative against PRRSV, *Mycoplasma hyopneumoniae* and PCV-2 at the beginning of the study [IDEXX PRRS X3 Ab Test, IDEXX Laboratorios, S.L., Barcelona, Spain; in-house PCR against *M. hyopneumoniae* (24) and PCV-2 (25)]. After a week of acclimatization period, 15 pigs were intranasally inoculated with 2 mL of porcine alveolar macrophages supernatant diluted in RPMI 1640 medium (Thermo Fisher Scientific, Barcelona, Spain) (control group). Twenty-five of the remaining piglets were intranasally inoculated with 2 mL of 10^5^ TCID_50_ of the low virulent PRRSV-1 3249 strain (subtype 1) (3249-infected group) (26) and the other 25 piglets were inoculated with 2 mL of 10^5^ TCID_50_ of the virulent PRRSV-1 Lena strain (subtype 3) (Lena-infected group) (27) under the same conditions. At 1, 3, 6, 8 and 13 days post-infection (dpi), 3 pigs from the control group and 5 from each infected group were humanely euthanized. Measurement of rectal temperature, clinical signs and abnormal behavior were monitored daily from one day prior to inoculation until the end of the study as described elsewhere (23). This experiment was approved by the IRTA Ethics Committee and by the Catalan Autonomous Government (Project 3647; FUE-2017-00533413) and carried out following the European Union guidelines (Directive 2010/63/EU). During the necropsies, thymus samples were collected and either immersed in TRIzol™ LS Reagent (Invitrogen, Carlsbad, CA, USA) and frozen at -80°C until further processing or fixated in 10% neutral buffered formalin and sectioned at 4 μm for the corresponding histopathological, histomorphometric and immunohistochemical studies.

### Histopathology and Histomorphometry of the Thymi

Histopathology and histomorphometry of the thymi have been previously reported (7). Briefly, for the histopathological and histomorphometric analyses, sections were stained with hematoxylin and eosin and Masson’s trichrome staining, respectively. The severity of the microscopic lesions from thymi was scored as follows: Grade 0, typical histopathological features of the thymus; Grade 1, focal cortical reduction; Grade 2, mild decrease of the ratio cortex/medulla (C/M), multifocal cortical reduction and evident tingible body macrophages; Grade 3, moderate decrease of the ratio C/M, multifocal cortical reduction with poor corticomedullary differentiation and an increase of the stroma, “starry sky” picture on the cortical layer; Grade 4, severe decrease of the ratio C/M, total disappearance of corticomedullary boundary distinction with an increase of the stroma and extensive presence of apoptotic bodies on the cortical layer.

### PCR Analysis

#### RNA Extraction and cDNA Synthesis

Total RNA was isolated from 100 mg of thymus, homogenized with 2 mL of TRIzol™ LS Reagent using a homogenizer150 (FisherBrand™ Thermo Fisher Scientific, Barcelona, Spain) and the NucleoSpin^®^ RNA virus columns kit (Macherey-Nagel, Düren, Germany) according to manufacturer’s protocols. To remove genomic DNA, a DNase type I Ambion^®^ TURBO-DNA-free™ kit (Life Technologies, Carlsbad, CA, USA) was applied. Concentration and purity of the extracted RNA were determined by spectrophotometry using the Nanodrop 2000 (Thermo Fisher Scientific, Barcelona, Spain), considering samples with a ratio 260/280 of about 2. The script™ cDNA Synthesis Kit (BioRad, Hércules, CA, USA) was used to generate cDNAs from 1 μl of total RNA as proposed by the manufacturer.

#### PRRSV Viral Load Analysis in Thymus

RNA was used for PRRSV genome quantification by LSI™ VetMAX™ PRRSV EU/NA 2.0 kit (Invitrogen), following manufacturer’s instructions. RT-qPCR reactions were performed in duplicate from 600 ng/μl from each sample by using the MyiQ™ 2 Two Color Real-Time PCR Detection System (BioRad) for 5 minutes (min) at 50°C, 10 min at 95°C followed by 40 cycles of 3 seconds (s) at 95°C and 30 cycles at 60°C for 30 s. An inter-run calibrator sample with a known quantification cycle (Cq) value was introduced in each plate to detect inter-run variations. To avoid overestimating the number of viral particles, results of PRRSV viral load in thymus were expressed in Cq as previously reported (28).

#### Relative Quantification of Immune Checkpoints

A relative quantification of the porcine immune checkpoints *PD1, PDL1, CTLA4, TIM3, LAG3, CD200R1* and *IDO1* was performed by using comparative *C_T_* method (also known as the 2^-ΔΔ^ *^C^* ^T^ method). Cq values of the above-mentioned target genes were normalized to the Cq values of the reference genes (29). *GeNorm* analysis (qbase+ 2.6.1 software, Biogazelle, Zwijnaarde, Belgium, www.qbaseplus.com) (30) was performed to determine the most stable reference genes from a set of 8 candidate reference genes and 10 representative cDNA samples. A screen of two reference genes with high stability (average *geNorm* M ≤ 0.5) were established as the optimal reference genes number. The optimal normalization factor was calculated with the arithmetic mean of the reference genes ribosomal protein L4 (*RPL4*) and hypoxanthine phosphoribosyltransferase 1 (*HPRT1*) (31). Sequences of the primers of reference genes and immune checkpoints are shown in **Table 1**. *PD1, PDL1* and *TIM3* primers were designed using the on-line *Primer3Plus* tool (www.primer3plus.com) (34). The iTaq™ Universal SYBR Green Supermix kit (BioRad) was used following the manufacturer’s instructions. Reactions were performed in triplicate by using 50 ng of cDNA from each sample and 0.5 µM of each primer in the MyiQ™ 2 Two Color Real-Time PCR Detection System (BioRad) for 20 s at 95°C for polymerase activation, followed by 40 cycles for melting (15 s, 95°C) and, annealing/extension (30 s, 60°C). After that, a melting curve analysis was performed (65–95°C) to verify the specificity of amplicons. An inter-run calibrator sample with a known Cq value was introduced in each plate to guarantee the quality of the retro-transcription and to detect inter-run variations. All PCR products were further verified by Sanger sequencing. Relative quantification results of porcine immune checkpoints from Lena-infected and 3249-infected animals were presented as fold change, comparing the value from each infected animal *versus* the average of control animals at each specific time point.

**Table 1:** Primer sequences of the porcine reference genes (RPL4 and HPRT1) and the different immune checkpoints genes (*PD1*, *PDL1*, *CTLA4*, *TIM3*, *LAG3*, *CD200R1* and *IDO1*).

*Genes*	*Type*	*Sequences*	*Reference*
*RPL4*	Reference gene	F 5´-CAAGAGTAACTACAACCTTC -3´ R 5´-GAACTCTACGATGAATCTTC -3´	(31)
*HPRT1*	Reference gene	F 5´-GGACTTGAATCATGTTTGTG-3-´ R 5´-CAGATGTTTCCAAACTCAAC-3´	(31)
*PD1*	Target gene	F 5´-AGCCCAAGCACTTCATCCTC-3´ R 5´-TGTGGAAGTCTCGTCCGTTG-3´	Designed
*PDL1*	Target gene	F 5´-GTGGAAAAATGTGGCAGCCG-3´ R 5´-TGCTTAGCCCTGACGAACTC-3´	Designed
*CTLA4*	Target gene	F 5´-TCTTCATCCCTGTCTTCTCCAAA-3´ R 5´-GCAGACCCATACTCACACACAAA-3´	(14)
*TIM3*	Target gene	F 5´-TTCGACGGGAGCAGTAAAGC-3´ R 5´-AGGGCAGGACACAGTCAAAG-3´	Designed
*LAG3*	Target gene	F 5´-CTCCTCCTGCTCCTTTTGGTT-3´ R 5´-CAGCTCCCCAGTCTTGCTCT-3´	(14)
*CD200R1*	Target gene	F 5´-TGTTCCAAGTTACTAATCAGGCTGAA-3´ R 5´-AGCCCATTAGCAACATGATACTCTTT-3´	(32)
*IDO1*	Target gene	F 5´-GGCACTTGATTGGTGGTCTC-3´R 5´-GCAATCCAAGCATCGTAAGG-3´	(33)

### Immunohistochemical Analysis

Immunohistochemical analysis of immune checkpoints was performed on thymus samples from control, 3249- and Lena-infected groups at selected time-points according to RT-qPCR expression results to identify the main cell subsets involved in their expression as well as their distribution within thymic structures (cortex and medulla). Thymus samples from pigs euthanized at 6, 8 and 13 dpi were submitted to immunohistochemical analysis against PD-L1 and CTLA-4, CD200R1 and TIM-3, respectively. EnVision FLEX Dual Link System-HRP (DAB+) (Dako, Burlingame, CA, USA) was performed for the detection of PD-L1 antibody and the Avidin-Biotin-Peroxidase Complex (ABC Vector Elite Laboratories, Burlingame, CA, USA) was performed for the detection of CTLA-4, TIM-3, and CD200R1 antibodies. Briefly, four µm tissue sections from thymus samples were dewaxed and rehydrated in a gradient of alcohols, followed by the endogenous peroxidase inhibition using EnVision FLEX peroxidase-blocking reagent (Dako) for 10 min in the case of PD-L1 and 3% H_2_O_2_ in methanol for 30 min in the case of CTLA-4, TIM-3 and CD200R1. As **Table 2** shows, different antigen retrievals were used for each antibody. After PBS washes (pH 7.4) and incubation with 100 µL of blocking solution, monoclonal primary antibodies against PD-L1, CTLA-4, TIM-3 and CD200R1 were applied and incubated overnight at 4°C in a humidity chamber. For the negative controls, the primary antibody was replaced by either an isotype control or by BSA to confirm the lack of non-specific binding. Thereafter, slides were washed with PBS and incubated with EnVision FLEX+ rabbit linker (Dako) for PD-L1 or the corresponding biotinylated secondary antibody (diluted 1:200 in 2% BSA) in the case of CTLA-4, TIM-3 and CD200R1 for 30 min at room temperature. Then, sections were incubated with EnVision FLEX/HRP (Dako) and Avidin-Biotin-Peroxidase Complex for 1 h at room temperature in darkness. Labeling was visualized by application of 3.3 diaminobenzidine chromogen (Dako) for PD-L1 and NovaRED™ substrate kit (Vector Elite Laboratories) for CTLA-4, TIM-3, and CD200R1. Lastly, sections were counterstained with Harris’ hematoxylin, dehydrated in graded ascending alcohols and xylene and mounted with Eukitt^®^ (Orsatec GmbH, Bobingen, Germany). Immunolabeled cells were identified and counted in 25 non-overlapping high magnification fields of 0.2 mm^2^ (Olympus BX51, Lympus Iberia SAU, L’Hospitalet de Llobregat, Barcelona, Spain) in each thymic structure (medulla and cortex). Identity between immunogens used to produce mAbs and porcine orthologues was 79.5% for PD-L1, 85.7% for CTLA-4 and 68.1% for TIM-3 (www.uniprot.org). Porcine CD200R1 mAb was developed by Poderoso et al. (20). Cross-reactivity of the used mAbs was defined by protein sequence homology and distinct staining on porcine cells. Immunohistochemistry against PD-1, LAG-3 and IDO-1 either was not successful or there was no available antibody which cross-react with porcine species.

**Table 2:** Summary of immunohistochemical methodology.

*Antibody*	*Clone*	*Commercial brand*	*Blocking solution*	*Dilution*	*Antigen retrieval*
PD-L1	E1L3N	Cell Signaling Tech., USA	3% BSA	1:200	Tris/EDTA pH 9^1^
CTLA-4	CAL49	Abcam, Cambridge, UK	2% BSA	1:500	Citrate pH 6^2^
TIM-3	4C4G3	Proteintech Group, Manchester, UK	2% BSA	1:50	Protease XIV
CD200R1	PCT3	In house, INIA	2% BSA	Neat	Protease XIV

BSA, bovine serum albumin. ^1^Tris/EDTA pH 9 pressure cooker treatment at 120°C for 5 min. ^2^Citrate pH 6, autoclave treatment at 121°C for 8 min. Protease XIV, enzymatic digestion (Sigma-Aldrich, USA) at 38°C for 6 min.

### Statistical Analyses

Differences between data of viral load in the thymus, the expression of the different immune checkpoints *PD1, PDL1, CTLA4, TIM3, LAG3, CD200R1* and *IDO1* in the thymus and immunohistochemical results against PD-L1, CTLA-4, TIM-3 and CD200R1 in the thymus were evaluated for approximate normality of distribution by the D’Agostino & Pearson omnibus normality test followed by the non-parametric Kruskal-Wallis test for multiple comparisons and the Mann Whitney’s U non-parametric test for unpaired groups. *P* value lower than 0.05 was considered statistically significant, indicated with ‘*’ (*P* ≤ 0.05) and ‘**’ (*P* ≤ 0.01).

Correlations between data of clinical score, temperature, microscopic lesion score, viral load and the expression of the different immune checkpoints in the thymus from 3249- and Lena-infected animals euthanized at 1, 3, 6, 8 and 13 dpi were assessed by the Pearson and Spearman tests and were considered relevant when *r* > 0.5 and *P* ≤ 0.05. Additionally, correlations between immune checkpoints and the expression of cell death markers (TUNEL, cCasp3, Fas, cCasp8, cCasp9 and iNOS) in the thymus from the same animals from a parallel study (7) were performed. Figures and data analyses were performed with GraphPad Prism 7.0 software (GraphPad Prism software 7.0, Inc., San Diego, CA, USA).

## Results

### Virulent PRRSV-1 Lena Strain Induced Higher Clinical Disease and Severe Thymus Atrophy

Clinical signs and histopathology were thoroughly described by our group (7, 23). Evident differences in the clinical score were observed between virulent Lena- and low virulent 3249-infected animals. Most Lena-infected piglets showed fever (above 40.5°C), together with severe dyspnea and tachypnea, apathy, postration and anorexia from 5 to 10 dpi, reflecting high clinical scores. In 3249-infected group, on the contrary, only few animals presented pyrexia and mild dyspnea as clinical signs [for details see (7, 23)].

**Figure 1:**
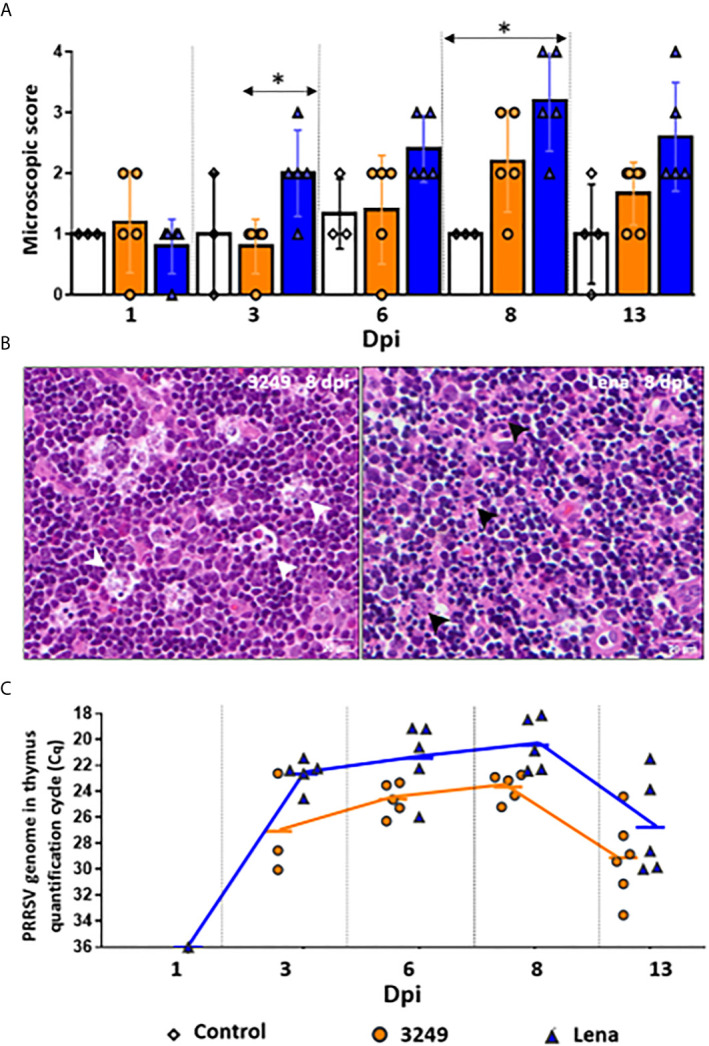
Microscopic findings and PRRSV viral load in thymus. Graph displays the microscopic score from thymi of each infected group (Grade 0-4). Columns represent mean ± SD. Significant differences between groups are represented (*P ≤ 0.05) **(A)**. Microscopic pictures of haematoxylin and eosin from a representative thymus of 3249-infected group euthanized at 8 dpi with evident tingible body macrophages (white arrows) in the thymic cortex (left, Grade 3) and Lena-infected group euthanized at 8 dpi with extensive apoptotic bodies (black arrows) in the thymic cortex (right, Grade 4) **(B)**. Graph displays PRRSV viral load in thymus of infected pigs along the infection. Values of PRRSV viral load are represented in quantification cycle (Cq). Lines represent mean **(C)**. Individual values for each animal from control (white diamond), low virulent 3249- (orange circle) and virulent Lena- (blue tringle) infected groups are represented.

Both infected groups showed progressive increase on the severity of the lesion, however, thymi from virulent Lena-infected animals showed significant differences in the microscopic score from 3 dpi onwards in comparison with low virulent 3249-infected animals (*P* ≤ 0.05) (**Figure 1A**). Whereas thymus from 3249-infected piglets were scored with Grade 2-3 (**Figure 1B**, left), thymus from Lena-infected piglets were scored with Grade 4 (8 and 13 dpi), which were characterized by elevated number of apoptotic bodies (**Figure 1B**, right), poor corticomedullar differentiation and severe decrease of the C/M ratio with an increase of the stroma, indicating severe thymic atrophy [for details see (7)].

### Virulent PRRSV-1 Lena Strain Caused an Earlier and Higher Viral Load in the Thymus Compared With Low Virulent 3249 Strain

Similar viral replication kinetics was observed in the thymus from virulent Lena- and low virulent 3249-infected animals, but the increase was earlier and higher in the former throughout the study (**Figure 1C**). PRRSV was detected as early as 1 dpi in the thymus of a pig from the Lena-infected group. At 3 dpi, the detection of PRRSV took place in the thymus from all Lena-infected animals and only in 3 out of 5 pigs from low virulent 3249-infected pigs. Both infected groups showed a peak of PRRSV replication at 8 dpi (Cq 23.10 ± 1.11 for 3249-infected animals and Cq 20.43 ± 2.04 for Lena-infected animals), dropping at 13 dpi in both infected groups (Cq 28.85 ± 3.28 for 3249-infected animals and Cq 26.76 ± 3.8 for Lena-infected animals). No statistical differences were found between both infected groups regarding PRRSV viral load in the thymus. PRRSV was not detected in control animals at any time-point

### Both Infected Groups Displayed an Up-Regulation of *PDL1* Gene at 6 dpi

**Figure 2:**
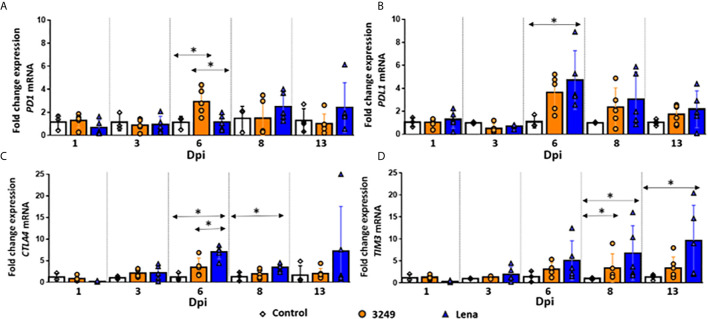
Relative mRNA expression of *PD1, PDL1, CTLA4* and *TIM3* mRNA. Graphs show fold change expression of *PD1* **(A)**, *PDL1* **(B)**, *CTLA4* **(C)** and *TIM3* **(D)** mRNA. Columns represent mean ± SD. Individual values for each animal from control (white diamond), low virulent 3249- (orange circle) and virulent Lena- (blue tringle) infected groups are represented. Significant differences between groups are represented (*P ≤ 0.05).

A similar trend was observed in the expression of *PD1* and *PDL1* in the thymus from low virulent 3249-infected piglets (**Figures 2A, B**). A considerable increase in the expression levels of both markers was observed at 6 dpi (fold change 2.93 ± 1.17 and 3.63 ± 1.60 for *PD1* and *PDL1*, respectively), showing significant differences with respect to control and virulent Lena-infected groups in *PD1* expression (*P* ≤ 0.05) (**Figure 2A**). Then, a progressive decrease until the end of the study was observed for both markers (fold change 1.07 ± 0.83 and 1.73 ± 0.82 for *PD1* and *PDL1*, respectively) (**Figures 2A, B**).

For virulent Lena-infected animals, kinetics of *PDL1* was similar to that observed in the thymus of low virulent 3249-infected animals, with a peak of expression at 6 dpi and a progressive decrease until 13 dpi, but always with higher values than those observed for 3249-infected pigs and control pigs (fold change 4.70 ± 2.55 at 6 dpi and fold change 2.17 ± 1.60 at 13 dpi) (**Figure 2B**). At 6 dpi, significant differences compared with control group were detected (*P* ≤ 0.05) (**Figure 2B**). However, in *PD1* marker this rise took place later, at 8 dpi (fold change 2.49 ± 1.26), keeping higher values compared with low virulent 3249-infected and control groups until the end of the study (fold change 2.41 ± 1.26 at 13 dpi) (**Figure 2B**).

**Figure 3:**
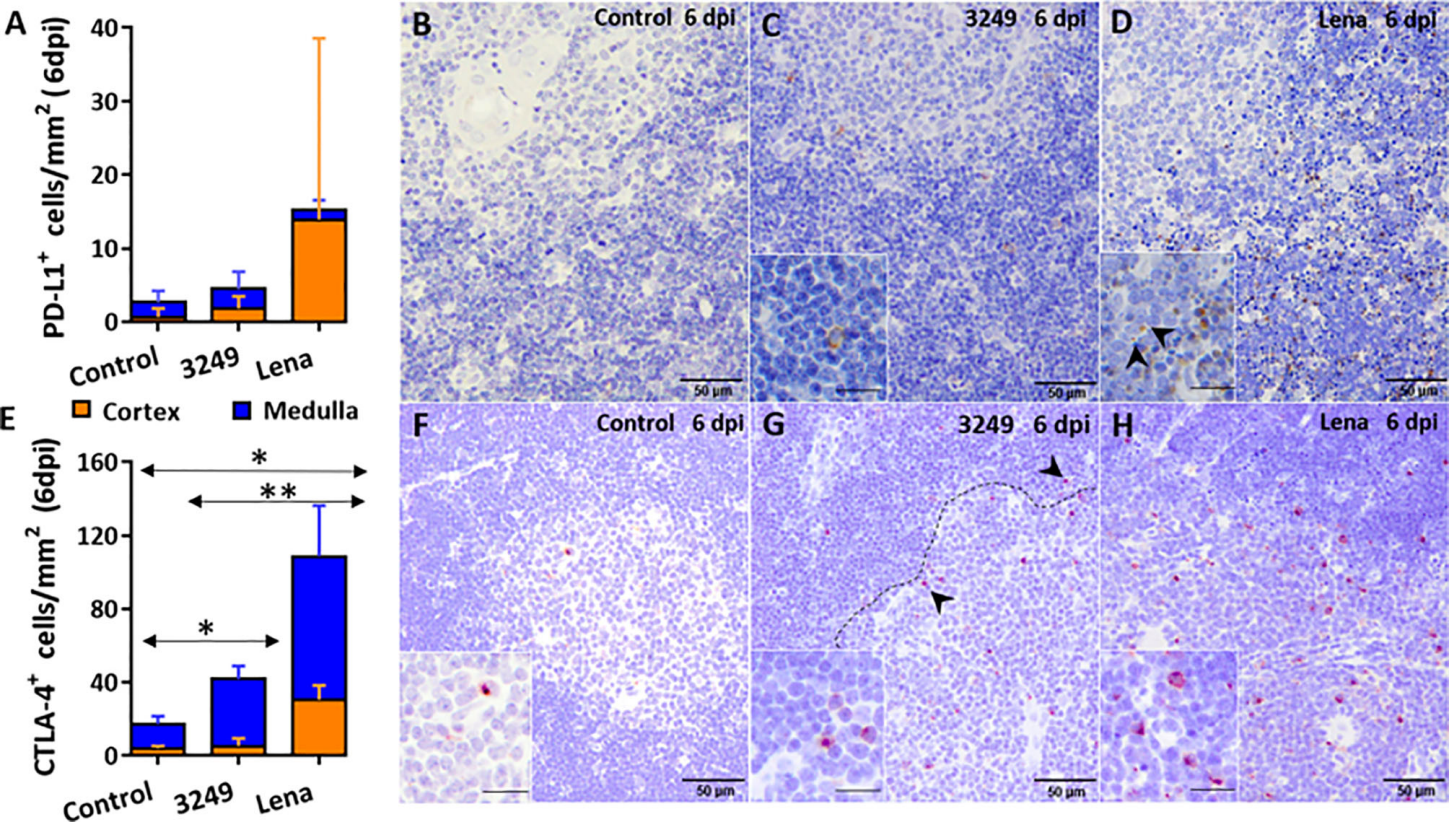
Immunohistochemical counting and expression of PD-L1 and CTLA-4 in the thymus of pigs euthanized at 6 dpi. Graph shows the number of PD-L1^+^ cells in the thymic cortex (orange) and medulla (blue) of pigs euthanized at 6 dpi **(A)**. Photomicrographs against PD-L1 in the thymus from a control pig **(B)**, from a pig infected with low virulent 3249 strain **(C)** and from a pig infected with virulent Lena strain **(D)** euthanized at 6 dpi. Insets show PD-L1*^+^* thymocytes in the thymic cortex of a 3249-infected pig and in apoptotic bodies of a Lena-infected pig (black arrows). Graph shows the number of CTLA-4^+^ cells in the thymic cortex (orange) and medulla (blue) of pigs euthanized at 6 dpi **(E)**. Photomicrographs against CTLA-4 in the thymus from a control pig **(F)**, from a pig infected with 3249 strain **(G)** and from a pig infected with virulent Lena strain **(H)** euthanized at 6 dpi. Dashed line shows corticomedullary boundary with CTLA-4^+^ thymocytes (black arrows). Insets show CTLA-4^+^ thymocytes in the corticomedullary boundary and the medulla of thymi from control and infected animals. Columns represent the mean of the number of immunolabeled cells expressed in cells/mm^2^ ± SD. Significant differences between groups are represented (*P ≤ 0.05) (**P ≤ 0.01).

Immunohistochemical labeling of PD-L1 revealed insignificant number of PD-L1^+^ cells in control group (**Figures 3A, B**). PD-L1 was detected in the cytoplasm of macrophage-like and DC-like cells of the medulla and thymocytes of the cortex in thymi from both infected groups (**Figures 3C, D**), but mainly in the thymic cortex from Lena-infected animals, associated with the extensive apoptosis pattern (**Figure 3D**).

### An Up-Regulation of the Gene *CTLA4* Was Observed at 6 dpi in Virulent PRRSV-1 Lena-Infected Group

An increase in the expression of *CTLA4* in the thymus of Lena-infected animals from 6 dpi (fold change 6.98 ± 1.57) was detected (**Figure 2C**), with statistically significant differences compared with both control group and low virulent 3249-infected group (*P* ≤ 0.05). Later on, a mild decrease in its expression was observed at 8 dpi, but still significantly up-regulated with respect to the control group (fold change for Lena-infected group 3.49 ± 0.86) (*P* ≤ 0.05). At 13 dpi, Lena-infected animals displayed a wide individual variability. For low virulent 3249-infected group the expression of *CTLA4* remained constant along the study (fold change around 2) with a small increase at 6 dpi (fold change 3.50 ± 2.10) (**Figure 2C**).

The immunolabeling of the CTLA-4 was mainly observed in the cytoplasm of thymocytes of the corticomedullary boundary and medulla, and scarcely in the cortex, from thymi of all groups (**Figures 3E–H**). Significant differences in the number of CTLA-4^+^ cells were detected between control and both infected groups (*P* ≤ 0.05), and between 3249- and Lena-infected groups (*P* ≤ 0.01) (**Figure 3E**).

### A Progressive Up-Regulation of *TIM3* Was Observed in Virulent PRRSV-1 Lena-Infected Group

A progressive up-regulation of *TIM3* was observed in thymi from virulent Lena-infected animals in comparison with control and low virulent 3249-infected groups from 1 to 13 dpi, increasing along the whole study (fold change 9.66 ± 7.98 at 13 dpi) and showing statistically significant differences at 8 and 13 dpi with respect to control group (*P* ≤ 0.05) (**Figure 2D**). In thymus from low virulent 3249-infected animals, a similar up-regulation of *TIM3* was observed at 6, 8 and 13 dpi, but in a lesser extent (fold change 3.10 ± 1.51; 3.39 ± 3.22, *P* ≤ 0.05; and 3.38 ± 2.53, respectively) (**Figure 2D**).

**Figure 4:**
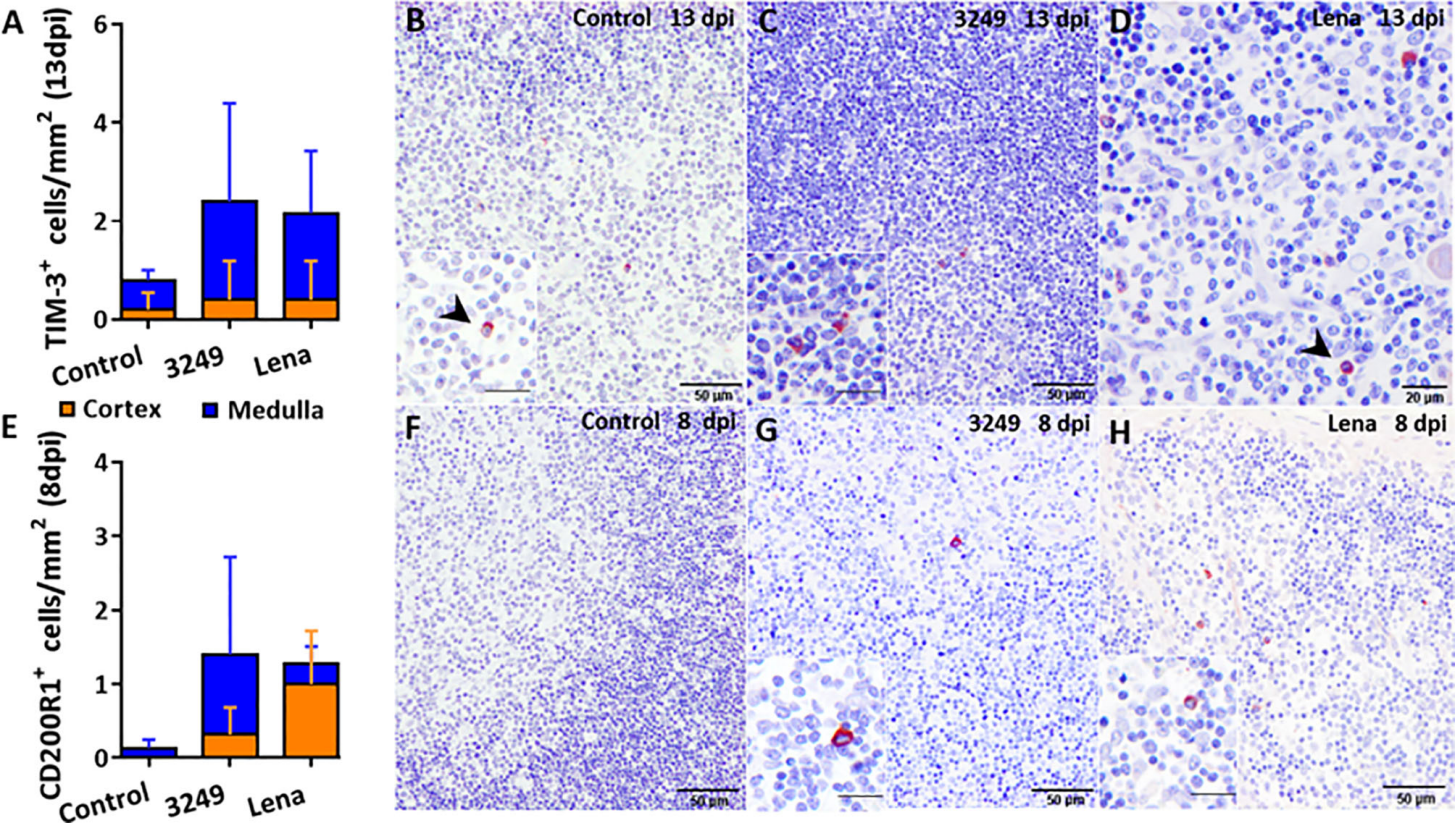
Immunohistochemical counting and expression of TIM-3 and CD200R1 in the thymus of pigs euthanized at selected time-points. Graph shows the number of TIM-3^+^ cells in the thymic cortex (orange) and medulla (blue) of pigs euthanized at 13 dpi **(A)**. Photomicrographs against TIM-3 from a control pig **(B)**, from a pig infected with low virulent 3249 strain **(C)** and from a pig infected with virulent Lena strain **(D)** euthanized at 13 dpi. Inset shows TIM-3^+^ macrophage-like cells in the medulla of the thymus from a 3249-infected pig. Black arrows show TIM-3^+^ thymocytes in the medulla from control and Lena-infected animals. Graph shows the number of CD200R1^+^ cells in the thymic cortex (orange) and medulla (blue) of pigs euthanized at 8 dpi **(E)**. Photomicrographs against CD200R1 in the thymus from a control pig **(F)**, from a pig infected with low virulent 3249 strain **(G)** and from a pig infected with virulent Lena strain **(H)** euthanized at 8 dpi. Insets show CD200R1^+^ macrophage-like cells in the medulla of thymi from infected animals. Columns represent the mean of the number of immunolabeled cells expressed in cells/mm^2^ ± SD.

Few number of TIM-3^+^ cells were detected in the thymus from all groups but were slightly higher in the thymus from both infected groups (**Figures 4A–D**). Immunostaining was detected in the cytoplasm of thymocytes and macrophage-like cells of the medulla (**Figures 4B–D**).

### Remarkable Up-Regulation of LAG3 Was Observed at 6 dpi in Both Infected Groups

**Figure 5:**
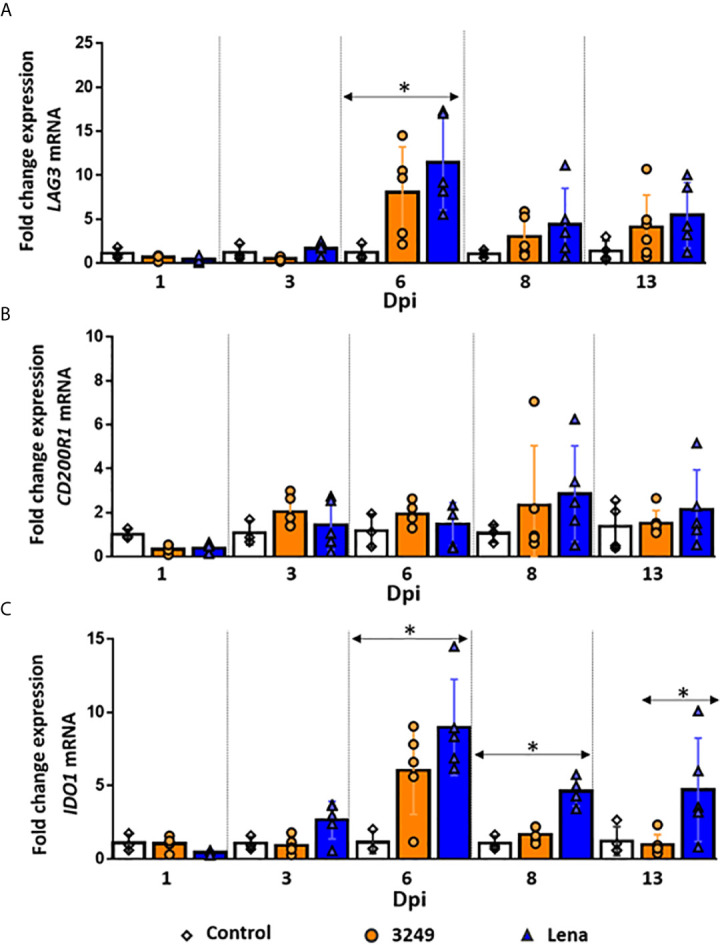
Relative mRNA expression of *LAG3*, *CD200R1* and *IDO1* mRNA. Graphs show fold change expression of *LAG3* **(A)**, *CD200R1* **(B)** and *IDO1* **(C)** mRNA. Columns represent mean ± SD. Individual values for each animal from control (white diamond), low virulent 3249- (orange circle) and virulent Lena- (blue tringle) infected groups are represented. Significant differences between groups are represented (*P ≤ 0.05).

A remarkable up-regulation in the expression of *LAG3* was observed at 6 dpi in the thymi of both infected groups, being stronger in the thymus of virulent Lena-infected animals (*P* ≤ 0.05) (fold change 11.44 ± 5.38 and 8.05 ± 5.15 for virulent Lena- and low virulent 3249-infected groups, respectively) (**Figure 5A**). At 8 dpi, the expression of *LAG3* drastically dropped in both infected groups (fold change 4.40 ± 4.11 and 3.03 ± 2.35, respectively) with a slight increase at 13 dpi (fold change 5.45 ± 3.72 and 4.10 ± 3.63 for virulent Lena- and low virulent 3249-infected groups, respectively) (**Figure 5A**).

### *CD200R1* Was Not Significantly Up-Regulated in the Thymus From Either Infected Group

A basal expression of the *CD200R1* gene was observed in the thymus of virulent Lena- and low virulent 3249-infected animals along the study (fold change around 1.50-2.00), but a mild increase, with a marked individual variability, was detected at 8 dpi in both groups (fold change 2.85 ± 2.17 and 2.33 ± 2.71 for Lena- and 3249-infected groups, respectively) (**Figure 5B**).

The number of CD200R1^+^ cells was scarce in the thymus from both infected groups and insignificant in the thymus from control group, detecting the labeling mainly in the cytoplasm of macrophage-like cells from medulla in 3249- and medulla and cortex in Lena-infected groups (**Figures 4E–H**).

### IDO1 Was Up-Regulated in Thymi From Virulent PRRSV-1 Lena-Infected Animals From 6 dpi Onwards

The expression of *IDO1* followed a similar kinetics in both infected groups, but a higher expression was observed in virulent Lena-infected animals from 3 dpi (**Figure 5C**). An up-regulation in the expression of *IDO1* was already detected at 3 dpi in the thymus from Lena-infected animals, followed by a considerable increase at 6 dpi (fold change 8.97 ± 3.28) and a decrease at 8 (fold change 4.62 ± 0.99) and 13 dpi (fold change 4.73 ± 5.52) (**Figure 5C**). At 6 and 8 dpi, significant differences were found with respect to the control group and at 13 dpi with respect to the low virulent 3249-infected group (*P* ≤ 0.05) (**Figure 5C**). In the thymus from 3249-infected animals, a peak of expression of *IDO1* was also observed at 6 dpi, but lower than in Lena-infected animals (fold change 6.05 ± 3.02), keeping basal levels at the remaining time points (**Figure 5C**).

### Correlation Study

**Figure 6:**
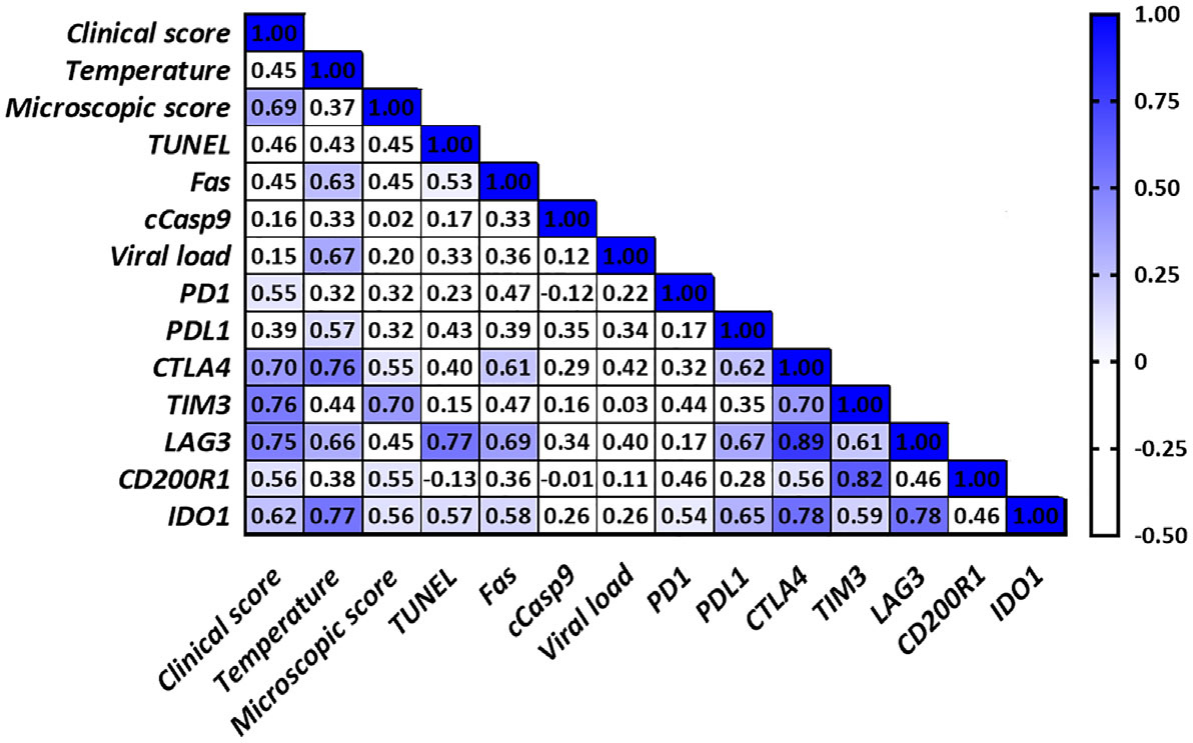
Correlations found in the thymus from virulent Lena-infected animals. Only significant correlations between clinical score, temperature, microscopic score, apoptosis markers, viral load and the different immune checkpoints in the thymus are indicated with blue color (*r* > 0.5 and *P* ≤ 0.05). Since no correlation was found between cCasp3, cCasp8 or iNOS and any of the parameters under study for any of both infected groups, these values were not included in the table for simplification purposes.

**Figure 7:**
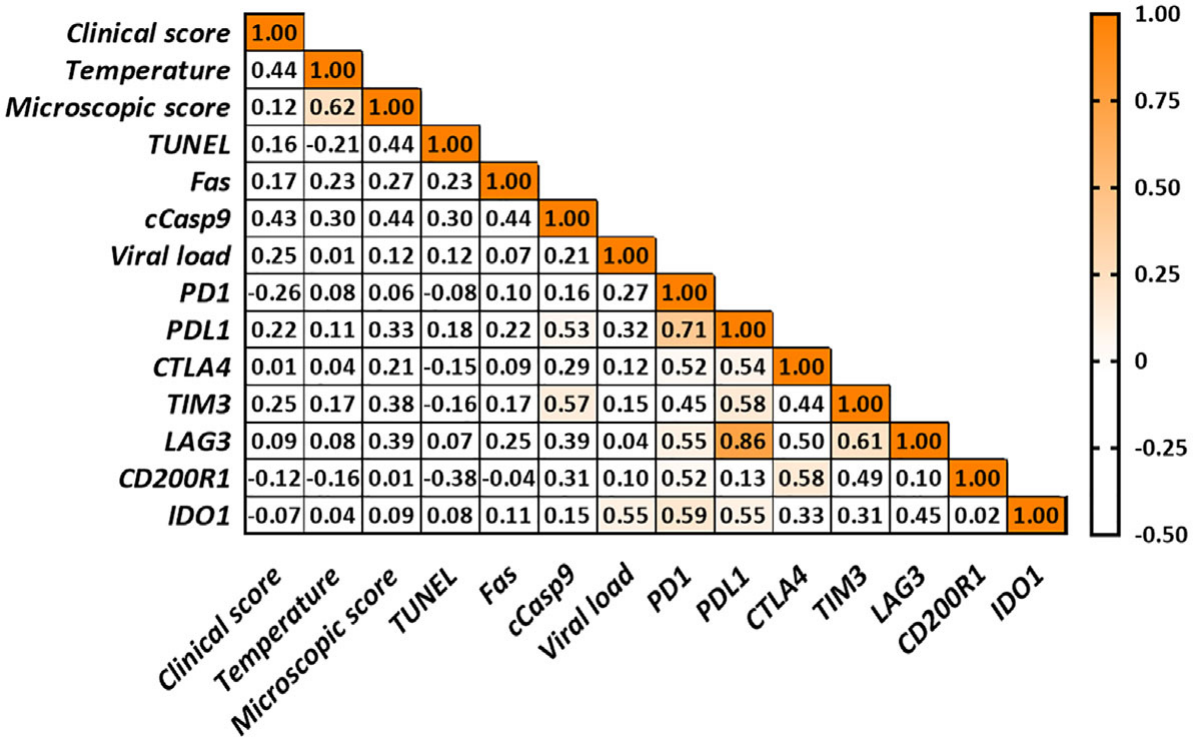
Correlations found in the thymus from low virulent 3249-infected animals. Only significant correlations between apoptosis markers, viral load and the different immune checkpoints are indicated with orange color (*r* > 0.5 and *P* ≤ 0.05). Since no correlation was found between cCasp3, cCasp8 or iNOS and any of the parameters under study for any of both infected groups, these values were not included in the table for simplification purposes.

Correlations found in the thymus from virulent Lena- and low virulent 3249-infected groups are summarized in **Figures 6** and **7**, respectively. Several correlations were found between the expression of the different immune checkpoints and clinical score, temperature, microscopic lesion score and several apoptosis markers (TUNEL and Fas) in the thymus from virulent Lena-infected animals (**Figure 6**) but no correlation between viral load and expression of immune checkpoints in the thymus were found. However, thymus from low virulent 3249-infected pigs showed a positive correlation between viral load and the immune checkpoint *IDO1* (*r* = 0.55). In this group, only cCasp9 apoptotic marker showed correlation with immune checkpoints in the thymus (**Figure 7**).

## Discussion

Virulent PRRSV strains have demonstrated possessing a higher thymic tropism than low virulent strains, leading to severe thymus atrophy, which could be related to marked immune dysregulation and susceptibility to secondary infections (6–8). T-cell dysfunction through immune checkpoints has been postulated as a strategy executed by PRRSV to subvert the immune response and escape from the host immune control (16); however, the role of immune checkpoints in a target lymphoid organ, such as the thymus, has not been studied in PRRS yet, particularly in animals infected with virulent PRRSV strains. In this study, we outline the expression of selected immune checkpoints (*PD1/PDL1*, *CTLA4*, *TIM3*, *LAG3*, *CD200R1* and *IDO1*) in the thymus of infected piglets during the early phase of PRRSV infection with strains of differing virulence. *PD1/PDL1*, *CTLA4*, *TIM3*, *LAG3* and *IDO1* immune checkpoints were significantly up-regulated in the thymus of PRRSV infected piglets, particularly in those infected with the virulent Lena strain.

PD-1 and CTLA-4 are both members of the CD28 co-stimulatory family and mediate T-cell exhaustion through the contact with their ligands, PD-L1/PD-L2 for PD-1 and CD80/CD86 for CTLA-4 (35–38). Whereas PD-1 can be expressed on T and B cells, natural killer (NK) T-cells, activated monocytes and DCs, CTLA-4 is mainly expressed on activated T-cells (36, 38). Upon IFN-γ stimulation, PD-L1 is expressed on several immune cells, such as T and B cells, NK cells, macrophages and myeloid DCs (37). This pattern of expression of PD-L1 coincides with the immunolabeling of PD-L1 observed in our study. PD-L1^+^ cells were thymocytes and apoptotic bodies from the cortex of the thymus from virulent Lena-infected animals and scattered macrophage- and DC-like cells from the medulla and cortex from both infected groups. We suggest that PD-L1^+^ cells could interact with CD80 from APCs, which would affect on positive selection during T-cell development in the thymic cortex, but also with PD-1 from thymocytes, leading the apoptosis of thymocytes.

In this sense, up-regulation of PD-1/PD-L1 axis and its association with apoptosis has been suggested in CSFV-infected pigs (14) as well as in *in vitro* experimental infections with some strains of PCV2 and PRRSV-2 and PCV2/PRRSV-2 co-infection (15, 16). Thus, PD-L1 immunolabeling was associated with extensive apoptosis pattern in the thymus from virulent Lena-infected pigs is in line with the activation of apoptosis through Fas/FasL pathway previously demonstrated in these animals by our group (7).

In the present study, an up-regulation of *PD1* and *PDL1* was detected in the thymus from low virulent 3249-infected animals at 6 dpi, with a strong correlation between both markers (*r* = 0.71), however, in case of virulent Lena-infected pigs, only *PDL1* showed a remarkable up-regulation at 6 dpi. Interestingly, PD-L1 may also interact with CD80, a ligand of CTLA-4 (39); thus, a higher expression of PD-L1 may accelerate the interaction between CD80 and PD-L1, interfering CD80 and CD28 binding and leading to the inhibition of T-cell activation, as previously suggested in PCV2-infected pigs (15). This mechanism may also represent a strategy of PRRSV to evade the host immune response in infected animals.

*CTLA4* was also overexpressed in the thymus from virulent Lena- but not in low virulent 3249-infected animals in our study. CTLA-4 binds with a higher avidity to the same ligands as CD28, namely costimulatory molecules CD80/CD86, interfering in T-cell development, proliferation and survival and suppressing negative selection at the thymus (35, 38, 40). CTLA-4 is also expressed in Tregs which may dampen the onset of an efficient host immune response by affecting the function and cytokine expression by T-cells, and hence, resulting in T-cell anergy (9, 41). Immunohistochemical expression of CTLA-4 was observed within the cytoplasm of thymocytes from the corticomedullary boundary and medulla, mainly in the thymus from virulent Lena-infected piglets. These CTLA-4^+^ T-cells could be migrating to the periphery, affecting on the T-cell activation in secondary lymphoid organs since co-ligation of TCR and CTLA-4 results in cell cycle detention and termination of T-cell activation (35, 38). Altogether, the up-regulation of *CTLA4* in our study points out a strain dependent mechanism involved in the modulation of the host’s adaptive immune response. Additional studies are required to determine if this finding could be linked with the virulence of PRRSV strains.

TIM-3 and LAG-3 are transiently up-regulated in activated CD4^+^ and CD8^+^ T-cells, in exhausted CD8 T-cells, Tregs, type 1 regulatory T cells and NK cells, but also in DCs in the case of TIM-3 (42). In our study, TIM-3 was mainly expressed in the cytoplasm of thymocytes and macrophage-like cells in the corticomedullary boundary and medulla in the thymus from virulent Lena-infected piglets. An up-regulation of *TIM3* and *LAG3* in the thymus from both PRRSV-infected groups was visible in our study, but significantly higher in thymi from Lena-infected pigs. TIM-3 negatively regulates Th1 CD4^+^ and CD8^+^ T-cells by inducing cell death upon interaction with galectin-9, its main ligand (43). The progressive increase in the expression of *TIM3* observed in the thymi from virulent Lena-infected pigs in our study correlated with the higher severity of lesions (*r* = 0.70) previously reported in thymi from these animals (7). On the other hand, *LAG3* exhibited evident differences in our study between control and infected groups, being significantly higher at 6 dpi in the case of Lena-infected group. A previous study with peripheral blood mononuclear cells from PCV2-infected pigs was not able to identify changes in the expression of LAG-3, suggesting a low participation of this marker during PMWS. LAG-3 binds to MHC class II competing with higher affinity than CD4 (19, 44), however, its impact on the function of CD8 and NK cells suggests the involvement of alternative ligands, such as LSECtin (42, 45), that could be expressed by thymic DCs (46). In this line, the expression of both TIM-3 and LAG-3 has been proposed to exert non-redundant synergistic effect on the impairment of CD8^+^ T-cell response together with the expression of PD-1/PD-L1 (42), which is supported by the high viral load and the correlation observed between these immune checkpoints in low virulent 3249- and virulent Lena-infected animals in our study.

CD200R1 is a transmembrane type I glycoprotein which interacts with CD200, inhibiting the expression of pro-inflammatory molecules, such as TNF-α, IFNs and inducible nitric oxide synthase (47). In contrast to human CD200R1, porcine CD200R1 is not expressed on T-cells, but it is expressed on alveolar macrophages and B cells (20, 48), such as the subset of thymic B cells (48), which may represent the findings observed in our study. However, no remarkable changes in the kinetics of *CD200R1* expression was observed in our experiment in the thymus from neither low virulent 3249- nor virulent Lena-infected piglets, ruling out any central role of this molecule at the thymus in the immunopathogenesis of the infection by these PRRSV strains.

IDO-1 is known to suppress T and B cells, NK cells, DCs and macrophages and generate and activate Tregs, as well as myeloid-derived suppressor cells (49). Pro-inflammatory mediators, such as IFN-γ and TNF-α, are potent inductors of IDO-1 expression by macrophages and DCs in several tissues which causes a depletion of tryptophan, the main substrate of IDO-1, and subsequent inhibition of T-cell proliferation (50, 51). A high expression of IDO-1 has been reported in CSFV-infected pigs (18) which in turn is able to modulate the host’s immune response (49). In our study, *IDO1* was highly up-regulated at 6 dpi in both infected groups but mainly in virulent Lena-infected pigs, coinciding with a peak of IFN-γ concentration in serum, as previously reported in a parallel study (52). An increase in TNF-α serum concentration has been also observed in the early phase of infection by Lena strain (53) as well as by other virulent PRRSV strains (54, 55). Interestingly, other immune checkpoints, such as PD-1/PD-L1, CTLA-4 and CD200R1, are associated with IDO-1 expression (49, 50), which is supported in our study by the correlation observed between *CTLA4* and *IDO1* (*r* = 0.78), between *PD1* and *IDO1* (*r* = 0.54) and between *PDL1* and *IDO1* (*r* = 0.65) in the thymus from virulent Lena-infected group.

Viral load and the length of antigen exposure during viral infections have been associated with the extent of T-cell exhaustion (11–13). Thus, the expression of PD-1 and its ligands PD-L1 and PD-L2 has been associated with the viral load in CSFV-infected pigs (14). In our study, higher viral load was detected in the thymus from animals infected with the virulent Lena strain, in line with previous results for PRRSV-2 virulent strains (56, 57). Although a positive correlation was only found between *IDO1* immune checkpoint and viral load in the thymus from low virulent 3249-infected piglets, the expression of most of the immune checkpoints was up-regulated from 6 dpi onwards when a high viral load was detected, with a higher fold-change in Lena- than in 3249-infected animals. Interestingly, increased frequencies of immune checkpoints have been associated with disease progression (9, 36, 42), also evident in our study through the correlations between various immune checkpoints and the clinical score, temperature and microscopic lesion score in the thymus from piglets infected with the virulent Lena strain (**Figure 6**). Therefore, the injured-thymus could induce the up-regulation of immune checkpoints in Lena-infected pigs as an attempt to control the underlying inflammatory response.

Different immune checkpoint co-expression has been related to the impairment of T-cell functions (9, 11) which in turn is correlated with apoptosis phenomena (11–13). In our study, correlations between different immune checkpoints and cell death markers in the thymus from virulent Lena-infected pigs were found (**Figure 6**). The co-expression of *PD1*/*PDL1*, *CTLA4*, *TIM3*, *LAG3* and *IDO1* highlight the onset of different inhibitory signals in the thymus between APCs and T-cells, especially in the thymus from Lena-infected group. This co-expression profile could affect T-cell development, maturation and selection, negatively impacting on T-cell population and its activation after antigen presentation in secondary lymphoid tissues. These features result in the impairment of the host immune response against PRRSV, hindering viral clearance and leading to persistent infection. Further studies should be conducted to determine the main subsets of cells involved in the expression of these molecules as well as synergic and complementary interactions among them.

## Data Availability Statement

The raw data supporting the conclusions of this article will be made available by the authors, without undue reservation.

## Ethics Statement

The animal study was reviewed and approved by IRTA Ethics Committee and by the Catalan Autonomous Government (Project 3647; FUE-2017-00533413) and carried out following the European Union guidelines (Directive 2010/63/EU).

## Author Contributions

IR-G, JG-L and LC conceived, designed and performed the project. FJ-P, IR-G and JG-L helped in the animal experiments and sample collection. IR-T, SG-L, JS-C, and FL-M made the laboratory experiments and analyzed the data. IR-T wrote the manuscript and IR-G and JG-L reviewed the manuscript. LC, FP and JG-L supervised the study and contributed to reagents/materials/analysis tools. All authors contributed to the article and approved the submitted version.

## Funding

JG-L is supported by a “Ramón y Cajal” contract of the Spanish Ministry of Economy and Competitiveness (RYC-2014-16735). This work was supported by the Spanish Ministry of Economy and Competitiveness (#AGL2016-76111-R and PID2019-109718GB-I00).

## Conflict of Interest

The authors declare that the research was conducted in the absence of any commercial or financial relationships that could be construed as a potential conflict of interest.

The handling editor declared a past co-authorship with several of the authors IR-T, FL-M, FP, LC, JG-L, IR-G, JS-C.

## Abbreviations

PRRSV, porcine reproductive and respiratory syndrome virus; dpi, days post-infection; DCs, dendritic cells; *PD1*, programmed cell death 1; *PDL1*, programmed cell death 1 ligand 1; CSFV, classical swine fever virus; PMWS, postweaning multisystemic wasting syndrome; PCV-2, porcine circovirus type 2; *CTLA4*; cytotoxic T-lymphocyte associated protein 4; *LAG3*, lymphocyte activating 3; *IDO1*, indoleamine 2,3-dioxygenase 1; *CD200R1*, CD200 receptor 1; C/M, cortex/medulla; min, minutes; s, seconds; Cq, quantification cycle; *RPL4*, ribosomal protein L4; *HPRT1*, hypoxanthine phosphoribosyltransferase 1; BSA, bovine serum albumin; NK, natural killer.
